# Can cluster analyses of linked healthcare data identify unique population segments in a general practice-registered population?

**DOI:** 10.1186/s12889-020-08930-z

**Published:** 2020-05-27

**Authors:** Kelechi Ebere Nnoaham, Kimberley Frances Cann

**Affiliations:** 1 Cwm Taf Morgannwg University Health Board, Ynysmeurig House, Navigation Park, Abercynon, Mountain Ash CF45 4SN UK; 2 University of Plymouth, Drake Circus, Plymouth, Devon PL4 8AA UK

**Keywords:** Population health, Population segmentation, Care utilisation, Cluster analysis

## Abstract

**Background:**

Population segmentation is useful for understanding the health needs of populations. Expert-driven segmentation is a traditional approach which involves subjective decisions on how to segment data, with no agreed best practice. The limitations of this approach are theoretically overcome by more data-driven approaches such as utilisation-based cluster analysis. Previous explorations of using utilisation-based cluster analysis for segmentation have demonstrated feasibility but were limited in potential usefulness for local service planning. This study explores the potential for practical application of using utilisation-based cluster analyses to segment a local General Practice-registered population in the South Wales Valleys.

**Methods:**

Primary and secondary care datasets were linked to create a database of 79,607 patients including socio-demographic variables, morbidities, care utilisation, cost and risk factor information. We undertook utilisation-based cluster analysis, using k-means methodology to group the population into segments with distinct healthcare utilisation patterns based on seven utilisation variables: elective inpatient admissions, non-elective inpatient admissions, outpatient first & follow-up attendances, Emergency Department visits, GP practice visits and prescriptions. We analysed segments post-hoc to understand their morbidity, risk and demographic profiles.

**Results:**

Ten population segments were identified which had distinct profiles of healthcare use, morbidity, demographic characteristics and risk attributes. Although half of the study population were in segments characterised as ‘low need’ populations, there was heterogeneity in this group with respect to variables relevant to service planning – e.g. settings in which care was mostly consumed. Significant and complex healthcare need was a feature across age groups and was driven more by deprivation and behavioural risk factors than by age and functional limitation.

**Conclusions:**

This analysis shows that utilisation-based cluster analysis of linked primary and secondary healthcare use data for a local GP-registered population can segment the population into distinct groups with unique health and care needs, providing useful intelligence to inform local population health service planning and care delivery. This segmentation approach can offer a detailed understanding of the health and care priorities of population groups, potentially supporting the integration of health and care, reducing fragmentation of healthcare and reducing healthcare costs in the population.

## Background

Globally, health care systems are increasingly interested in population health. In many developed countries, improvements in life expectancy have slowed or stalled and health inequalities are increasing [1]. Population health as an approach seeks to improve physical and mental health outcomes, promote wellbeing and reduce health inequalities across whole populations. Growing interest in population health is possibly due to recognition of the challenges facing health care systems – rising costs, ageing populations, unhealthy lifestyle choices and deepening poverty in society [2]. These challenges lend themselves to explanatory and interventional models inherent in the population health approach. At the core of this approach is the goal of improving health outcomes for whole populations – not just for those seeking care – while paying attention to the distribution of those outcomes within the population [3].

One of the key pillars of population health is person-centred integration of health and care systems, a reflection of the need to reduce fragmentation of care around the growing numbers of patients with multiple long-term conditions [1]. Person-centred care is however not feasible if, in population health policy terms, it implies developing care pathways unique to every individual in the population [4]. Population segmentation, which involves grouping populations on the similarity of one or more proxies of health needs, potentially allows definition of population groups for whom integrated and tailored health and care interventions across the continuum of care can be tailored [5].

Two broad approaches to population segmentation have evolved in recent years. In traditional (or expert-driven) approaches, a population is segmented on a-priori, expert-defined criteria informed by literature review and consensus [6]. For example, the Suicide and Self Harm Prevention Strategy for Wales 2015–2020 highlights the need to focus preventative efforts towards men aged 15–44 years [7]. In England, the London Health Commission segmented the population of London based on morbidity and age group [8]. This approach is limited by lack of generally agreed ways of: (i) knowing the number of natural clusters in the population, and (ii) determining the variables on which to base segmentation. Furthermore, grouping populations on criteria, such as age and morbidity, does not accurately reflect actual use of health and care services.

More recently, population segmentation based on health and care utilisation has gained recognition as an alternative. This data-driven segmentation approach potentially generates detailed insight into the needs of populations using a variety of analytical methods applied to large integrated datasets from various health and care settings [9]. A recent study exploring this approach was limited by failure to include data on use of A&E care [10]. In addition, it was based on a random selection of General Practice-registered patients across England and therefore did not reflect local patterns, a critical component of local health and care planning and service delivery.

This study therefore set out to explore the potential for using utilisation-based cluster analyses to segment a local GP-registered (and geographically-defined) population in the South Wales Valleys. This was done in two sequential steps – first assessing whether utilisation-based cluster analyses could identify clusters of patients in the population based on healthcare utilisation parameters and, secondly, undertaking detailed profiling of the utilisation-based segments to indicate their healthcare needs [11].

## Methods

### Data

We created a pseudonymised integrated dataset by linking, at a patient-level, primary and secondary care data for a population of about 80,000 people registered with General Practices in one geographical locality of the South Wales Valleys. For each patient, we identified seven healthcare utilisation variables on which cluster analyses were based – elective inpatient admissions, non-elective inpatient admissions, outpatient first attendances, outpatient follow-up attendances, A&E attendances, GP visits (specifically those for which a General Practitioner was seen) and count of distinct drugs used in the year. We selected these seven utilisation variables because they reflected different types of healthcare providers and use of health care resources across different parts of a health care system [10]. Five of these variables have previously been identified as suitable for data-driven utilisation based segmentation across healthcare providers without overlap [10]. The number of outpatient attendances was further broken down into first attendance and follow-up attendance, and the number of A&E attendances was included, as we felt that these offered additional understanding of the healthcare needs of our population. We also included data on patient characteristics such as long-term condition (LTC) diagnoses, age, deprivation, smoking status, cost and scores for risk of emergency admission in the next 12 months.

### Cluster analyses

We carried out sequentially two types of cluster analyses on the dataset. We conducted hierarchical cluster analysis which allows identification of the optimal number of clusters in the population as readily available stopping rules mean it does not require a priori selection of number of clusters [12]. Given that hierarchical cluster methods are sensitive to outliers and are generally not suitable for larger datasets, [13] we followed this with k-means non-hierarchical cluster analysis used with an Euclidean distance. This method is efficient and can handle large datasets [14].

Hierarchical cluster analysis was conducted by selecting 10 random population subsets of size 3000 and calculating the pseudo F-statistic defined by Calinski and Harabasz [15]. This approach assessed the cluster tightness for increasing cluster size (2 to 20) by comparing the mean sum of squares between groups to that within groups. The pattern, which was of a gentle decline in the pseudo F-statistic seen almost consistently across the 10 subsets, did not clearly suggest an optimal number of clusters. The Duda and Hart Je (2)/Je (1) index [16] was then calculated. This used the within-cluster sum of squared distances from the mean to compare the present cluster to a potential further split. The suggested rule of thumb for deciding on the number of clusters is to look for a clustering solution with a high Duda-Hart and a corresponding low pseudo T-squared value, with high pseudo T-squared values on either side [17]. Using this method we determined that the optimal number of clusters was approximately 10. K-means analysis for the entire dataset population was then performed to create the final 10 clusters for the population.

All clustering was done on standardised versions of the 7 healthcare utilisation variables derived by subtracting the mean of each variable and dividing by its standard deviation. This ensured that each variable got equal weight in the determination of “distance” used by the various clustering methods.

All cluster analysis was done in Stata 15 [18].

### Statistical analyses and cluster profiling

The clusters (hereafter referred to as segments) were then assessed and profiled on the average of the healthcare utilisation variables, as well as other characteristics such prevalence of LTCs, age, deprivation and risk of emergency hospital admission in the next 12 months. The statistical analyses sought to determine whether there were statistically significant differences across the segments in each characteristic. For the mean counts of healthcare utilisation variables and number of LTCs, we used a Kruskal-Wallis test of differences of means as these variables did not meet Normality assumptions. For age and risk of emergency admission score, an ANOVA test for difference of means was estimated. For the proportions of the population who were smokers and who were in the most deprived population quintiles, as well as for segment prevalence of LTCs, we calculated Chi square tests for proportions. The variables which differed significantly in the statistical tests of difference were then explored pair-wise between segments using Mann–Whitney U tests (for the non-Normal continuous variables), Student t-tests (for the Normal continuous variables), and z-tests (for the categorical variables). We adjusted the significance level of 0.05 for the pair-wise tests using the Bonferroni method to account for multiple testing done in comparison of the segments.

The determinants of healthcare need and healthcare complexity differ [19, 20]. Therefore, in profiling segments, we applied a rule of thumb that distinguished these, defining ‘high need’ segments as ones fulfilling either of two criteria: (i) mean activity (count) more than 100% above the mean for the study population in any care setting, or (ii) mean activity (count) more than 20% above the mean for the study population in 4 or more care settings. Segments were identified as ‘high complexity’ if they had mean activity (count) higher than the mean for the study population in 4 or more care settings.

## Results

The study population included 79,607 patients (50.1% Female) with an average age of 41.4 years. All patients were registered with the General Practices in the Rhondda locality of Cwm Taf Morgannwg in the South Wales Valleys. K-means cluster analysis produced ten segments based on healthcare utilisation patterns across diverse settings of health care provision (Table 1). All seven healthcare utilisation variables were statistically different across the segments - reflecting the central aim of cluster analysis which is to maximise the distance between clustering variables. In addition, the non-clustering variables – patient characteristics and much LTC prevalence – were also found to differ significantly, demonstrating that each segment was largely unique.

**Table 1:** Characteristics of the Segments

	Segments											ANOVA or Kruskal- Wallis/χ2 test
	1	2	3	4	5	6	7	8	9	10	Population value	
**Care Utilisation, mean (SD)**												
Non-elective inpatient admissions	0.01 (0.1)^z^	0.17 (0.41)^x^	1.27 (0.54)^x^	3.27 (1.68)^x^	0.02 (0.15)^y^	0.34 (0.55)^x^	0.35 (0.69)^x^	0.02 (0.13)	1.25 (1.29)^x^	0 (0)^z^	0.14 (0.52)	KW: < 0.000
Elective inpatient admissions	0 (0)	1.22 (0.54)^x^	0.03 (0.16)^x^	0.36 (0.65)^x^	0 (0)	0.14 (0.36)^y^	0.1 (0.37)^y^	0 (0)	0.14 (0.43)^z^	0 (0.02)	0.08 (0.34)	KW: < 0.000
Outpatient first attendances	0 (0)^x^	0.87 (0.88)^y^	0.39 (0.6)^x^	1.79 (1.82)^x^	1.23 (0.52)^y^	1.89 (1.19)^y^	1.26 (1.17)^y^	0.17 (0.39)^x^	0.92 (1.13)^y^	0.09 (0.3)^x^	0.34 (0.72)	KW: < 0.000
Outpatient follow-up attendances	0.14 (0.71)^x^	2.02 (2.39)^x^	1.17 (1.85)^y^	4.22 (4.69)^y^	1.17 (1.92)^y^	3.66 (3.03)^y^	20.53 (12.98)^x^	0.79 (1.52)^x^	2.26 (3.96)^x^	0.26 (0.92)^x^	0.91 (2.98)	KW: < 0.000
GP practice visits	0.11 (0.4)^x^	1.34 (1.56)^y^	1.3 (1.28)^y^	4.94 (3.54)^x^	0.93 (1.06)^x^	4.6 (2.29)^x^	1.55 (2.05)^z^	0.71 (1.02)^x^	7.55 (3.28)^x^	0.81 (0.99)^x^	0.74 (1.51)	KW: < 0.000
Prescribing (Distinct Drug Count)	1.78 (2.04)^x^	8.11 (6.39)^y^	6.65 (5.64)^x^	15.9 (10.01)^z^	4.31 (3.61)^x^	15 (7.92)^z^	8.28 (7.62)^y^	12.35 (4.58)^z^	20.64 (9.23)^x^	2.61 (2.77)^x^	4.75 (5.8)	KW: < 0.000
A&E attendances, mean (SD)	0 (0)^x^	0.48 (0.81)^x^	1.52 (1.02)^y^	5.13 (3.15)^x^	0.28 (0.49)^x^	1.13 (1.22)^x^	0.69 (1.12)^x^	0.14 (0.39)^x^	2.04 (1.93)^y^	1.46 (0.84)^x^	0.41 (0.95)	KW: < 0.000
**Patient characteristics**												
Age, mean (SD)	36.2 (21.5)^x^	53.6 (20.5)^x^	38.2 (27.9)^x^	47.5 (27.3)^x^	42.9 (23)^y^	59.3 (21.4)^y^	42.1 (20.3)^y^	60.4 (18.5)^y^	82.1 (11.1)^x^	29.8 (20)^x^	41.4 (23.7)	AN: < 0.000
Quintile 1 & 2 Deprivation, %	84.2	85.7	87.6	88.6	85.0	85.2	86.5	86.1	78.5^z^	85.6	85.0	χ2: < 0.000
Current smoker, %	20.3	23.1	22.3	27.2	22.1	22.1	28.0	25.9^z^	12.8^x^	20.6	21.6	χ2: < 0.000
Risk of emergency admission in next 12 months, mean (SD)	0.07 (0.05)^x^	0.27 (0.13)^x^	0.23 (0.14)^y^	0.45 (0.19)	0.2 (0.1)^y^	0.38 (0.15)	0.36 (0.16)^z^	0.24 (0.12)^x^	0.5 (0.17)^z^	0.12 (0.08)^x^	0.15 (0.13)	AN: < 0.000
**Long Term Conditions (LTCs)**												
Number of LTCs, mean (SD)	0.58 (0.92)^y^	2.55 (2.36)^x^	2.08 (2.44)^x^	5.27 (4.03)^y^	1.22 (1.46)^x^	3.75 (2.68)^y^	2.31 (2.45)^x^	2.89 (1.95)^x^	6.46 (3.39)^x^	0.64 (1)^y^	1.32 (1.9)	KW: < 0.000
Long Term Condition, prevalence in %												
ARMD	0.2^y^	1.6	0.9^z^	1.9	0.7^z^	2.8^z^	2.2	1.9	9.0^x^	0.2^z^	0.7	χ2: < 0.000
Arthritis	0.2^z^	1.2	1.2	2.6^z^	0.8	5.0^y^	1.2	2.4^z^	5.7^y^	0.3^z^	0.9	χ2: < 0.000
Asthma	7.1^y^	15.7	13.6	24.6^z^	10.3^z^	21.3^z^	16.2	21.9^z^	11.8	9.6^z^	11.0	χ2: < 0.000
Bipolar Disorder	0.1^z^	0.4	0.5	2.7^z^	0.4	1.3^z^	2.5^z^	0.8	1.4	0.1	0.4	χ2: < 0.000
CHF	0.1^z^	1.8^z^	2.1	8.4^x^	0.5^z^	4.8^x^	2.2	2.6^z^	14.9^x^	0.1^z^	1.0	χ2: < 0.000
COPD	0.7^y^	7.1^z^	6.3^z^	20.6^x^	2.0^x^	12.9^y^	6.5^z^	12.2^y^	30.7^x^	0.5^y^	3.6	χ2: < 0.000
CRF	1.1^y^	6.7^z^	5.7^z^	15.7^y^	3.2^y^	13.1^y^	5.6	10.8^x^	31.0^x^	0.8^y^	3.8	χ2: < 0.000
Depression	6.8^x^	15.4	13.5	28.7^y^	11.9^y^	17.7	17.2	16.8	22.0	8.5^x^	10.3	χ2: < 0.000
Diabetes	2.1^y^	11.7^y^	8.2^x^	21.7	5.3^x^	25.4	13.3^y^	24.9	24.8	1.9^y^	7.1	χ2: < 0.000
Glaucoma	0.5^y^	3.4	1.5	2.8	1.3	4.5^y^	2.0	3.2	9.5^x^	0.3^y^	1.3	χ2: < 0.000
Hypertension	9.5^x^	33.4^x^	22.8^y^	39.2^x^	17.8^y^	46.6^x^	21.0^z^	49.5^x^	68.1^x^	7.2^x^	18.9	χ2: < 0.000
Hyperthyroidism	1.8^y^	7.4^y^	4.9^z^	11.1^z^	4.1^z^	11.7^z^	5.2	11.5^z^	17.5^x^	1.6^y^	4.2	χ2: < 0.000
Ischaemic Heart Ds	0.6^y^	7.6^z^	6.8^z^	19.8^y^	2.6^y^	17.5^y^	5.1	11.7^x^	30.0^x^	0.8^y^	3.8	χ2: < 0.000
Low Back Pain	1.1^y^	5.1	3.6	8.8^y^	2.2^z^	5.8^z^	2.8	4.4	5.4	2.0^z^	2.3	χ2: < 0.000
Osteoporosis	0.0^z^	0.8^z^	1.4^y^	5.8^y^	0.3	2.1^x^	0.7	0.4	7.1^y^	0.0	0.4	χ2: < 0.000
Parkinson’s Ds.	0.0	0.1	0.4	1.1	0.3	1.1^y^	0.2	0.5	5.0^x^	0.0	0.2	χ2: < 0.000
Schizophrenia	0.4^z^	0.8	1.6^z^	4.6^y^	0.9^z^	2.3^y^	10.7^x^	1.3	4.5^y^	0.3^z^	0.9	χ2: < 0.000
Seizure Disorder	0.8	2.1^z^	4.0^z^	10.4^x^	1.2	3.4	2.2	3.0	4.7	1.3	1.6	χ2: < 0.000

Significantly different from all 9 other segments; y: Significantly different from 8 other segments; z: Significantly different from 7 other segments; All at 0.05/9 = 0.0056 significance level (Bonferroni adjustment). All variables are significantly different across segments at a < 0.000 significance level using ANOVA, Kruskal-Wallis or Chi Square tests; ARMD – Age-Related Macular Degeneration; CHF – Congestive Heart Failure; COPD – Chronic Obstructive Pulmonary Disease; CRF – Chronic Renal Failure

There was significant deprivation in the population, with 85% of people living in the two most deprived national quintiles. The prevalence of current smoking in the study population was 21.6% - a figure consistent with rates reported for the region [21]. The average number of LTCs per person was 1.32 but this ranged from 0.6 to 6.5, highlighting the tendency toward multiple morbidity in this population. The commonest LTCs in the study population were asthma (11%), depression (10.3%), diabetes (7.1%) and hypertension (18.9%). These rates, were again largely consistent with those reported for the same population [22].

### Profiling the population segments

**Fig. 1:**
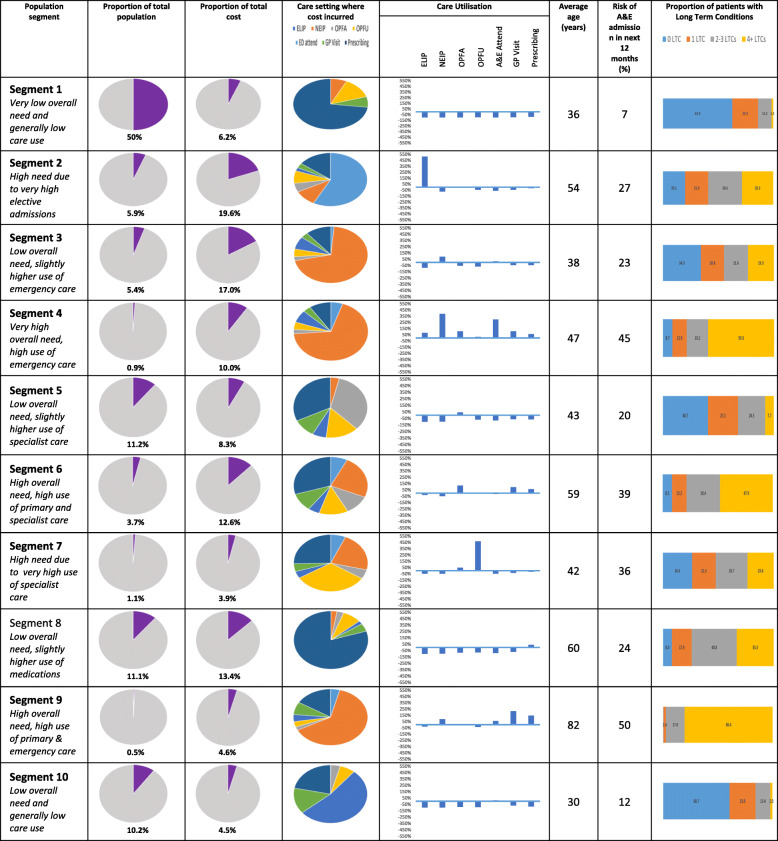
Profiles of Segments

For each segment, specific attributes are presented in comparison with the average for the study population (Table 2 & Fig. 1).

**Table 2:** Further Characteristics of Segments

	Segment 1	Segment 2	Segment 3	Segment 4	Segment 5	Segment 6	Segment 7	Segment 8	Segment 9	Segment 10
**Characterisation**	Low Need, Low Complex	High Need, Low Complex	Low Need, Low Complex	High Need, High Complex	Low Need, Low Complex	High Need, High Complex	High Need, Low Complex	Low Need, Low Complex	High Need, High Complex	Low Need, Low Complex
**Moderate-Severe Frailty (%)**	0.4%	12.9%	9.5%	28.3%	4.0%	32.3%	11.8%	20.9%	66.0%	1.0%
**Average Bed Days in 2017**										
**Elective**	0.00	0.64	0.02	0.21	0.00	0.10	0.07	0.00	0.46	0.00
**Non-elective**	0.00	0.35	3.2	11.1	0.07	2.00	1.76	0.06	11.9	0.00
**Maternity**	0.01	0.01	0.04	0.14	0.05	0.01	1.26	0.01	0.00	0.01
**Number of people**	39,821	4720	4274	739	8899	2950	851	8836	423	8094
**Total cost (£m)**	3.2	10.0	8.7	5.1	4.2	6.4	2.0	6.8	2.3	2.3
**Cost per head of population**	£79	£2121	£2025	£6912	£476	£2181	£2315	£772	£5489	£285
**Cost to population ratio**	0.12	3.32	3.15	11.11	0.74	3.41	3.55	1.21	9.20	0.44
**Predominant LTC**	None	None	None	Most major LTCs	None	Asthma, arthritis, CRF, DM, HT, IHD	Bipolar disorder, schizophrenia	Asthma, DM, HT & IHD	Most major LTCs	None

Although Segments 1, 3, 5, 8 and 10 were characterised broadly as ‘low need, low complexity’ segments, there were notable differences in their profiles. Segments 1 (mean age 36 years) and 10 (mean age 30 years) were on average young adults with 0–1 LTC, whose healthcare utilisation profiles were low in all the healthcare settings assessed. In addition, about half of the study population, despite the general high levels of deprivation, were in Segment 1 – with few and low-complexity healthcare needs. Segments 3 and 5 were on average young and middle-aged adults with 1 or 2 LTCs. For these segments, no specific LTCs were dominant in terms of prevalence. Segment 3 may include individuals with suboptimal control of LTCs, resulting in higher than average use of non-elective inpatient care – and consequent high per capita cost - although this did not reach the ‘high need’ threshold of our rule of thumb. Despite their similarities with Segment 3, Segment 5 patients have a much lower cost per capital profile. This probably reflects the impact of lower-than-average use of non-elective inpatient care associated with having fewer LTCs (1.2, cf. 2.1 in Segment 3) that are also probably better-managed through appropriate outpatient care and prescribing. The Segment 8 population is an older adult population (mean age 60 years) with an average of 3 LTCs per person – predominately ambulatory care sensitive (ACS) conditions - asthma, diabetes, hypertension and Ischaemic Heart Disease (IHD). Their higher-than-average use of prescribing possibly reflects success in ACS condition management.

Segments 2 and 7 were characterised broadly as ‘high need, low complexity’ segments. Segment 2 patients were older adults (mean age 54 years) with particularly high use of elective inpatient care. Although they have 2–3 LTCs on average, there is no dominant LTC that might be driving elective inpatient care use. As elective care is the standard route for many common operations [23], elective surgery may account for the high per capita cost from elective inpatient care use in this segment. Segment 7 patients similarly have high per capita cost consumption attributable to high utilisation of care in one setting – in this case outpatient follow-up visits. The dominance of bipolar disorder and schizophrenia in this segment suggests these mental health disorders may be driving outpatient follow-up care use in this segment. COPD, Diabetes and Hypertension were relevant LTCs that may have contributed to use of non-elective inpatient care and prescribing in this segment. Segment 7 patients also had the highest number of maternity bed days during the year of all Segments.

Segments 4, 6 and 9 are the ‘high need, high complexity’ segments in this population and their diversity in age (47, 59 and 82 years, respectively) and high per capita cost underscore the fact that significant and complex healthcare need is a feature across age groups in this population. Segment 4 makes up only 1% of the population but accounted for 10% of total healthcare expenditure. It has the highest proportion of people living in the 2 most deprived quintiles, the highest cost: population ratio and is one of three segments with the highest prevalence rates of current smoking. High healthcare consumption is consistent across all settings and probably reflects the average > 5 LTCs per person. Most major LTCs are significantly prevalent, comparatively, in this segment but the types of conditions also reflects their younger age (seizure disorders, bipolar disorder, low back pain, depression and asthma). This contrasts with the significantly less deprived and older patients of Segment 9 whose predominant LTCs included arthritis, Chronic Renal Failure, Age-Related Macular Degeneration, Glaucoma and Parkinson’s disease. The average of 6.5 LTCs in Segment 9 perhaps explains their high frequency of use of primary care, prescribing and emergency hospital care.

## Discussion

A central goal of population segmentation is to identify population subgroups that are homogeneous enough in terms of healthcare needs to enable tailoring of integrated health and care for them [24]. This study demonstrates that cluster analyses of linked healthcare data can identify distinct segments of care users in a local General Practice-registered population. Further profiling of the segments in the study population established that they had unique demographic and morbidity attributes that could potentially support planners and providers of health and care services in responding more accurately to the needs and priorities of each segment [4].

‘Low need’ population groups tend not to be prioritised by health systems as they make relatively little demand on services. They may therefore not be deemed ‘impactable’ but proactively availing them of preventative services is critical to healthcare sustainability. To enable tailoring of preventative care to ‘low need’ populations, further differentiation across this often-sizeable segment of the population is necessary. Although we found that nearly 88% of our study population were in ‘low need’ segments, our approach to segmentation demonstrated the heterogeneity in this group with respect to age, morbidity, per capita cost of care, settings in which care was mostly used and prevalence of relevant risk factors. While Segments 1 and 10 might benefit from targeted and universal preventative initiatives to support them in staying healthy and non-care-seeking, Segment 3 patients could be targeted with hospital-based preventative services, such as smoking cessation [25], and improved LTC management to reduce emergency hospital admissions. The high prevalence rates of current smoking in Segment 8 patients, combined with prevalent ACS conditions and a higher-than-average use of prescribing, indicates they could be an ideal segment for integrated approaches involving active ACS condition management, lifestyle risk modification and medication reviews.

The ‘high need, low complexity’ Segments have high per capita cost incurred in a restricted setting (elective inpatient care in Segment 2 and outpatient follow-up care in Segment 7). For these Segments, understanding and de-escalating need quickly is key. The Segment 7 population has a relatively high prevalence of mental health disorders but outpatient visits during the 30 days after a mental health hospital discharge are reported to be associated with a lower hospital readmission risk [26]. Consequently, improving care in this population segment may require alternative closer-to-home models of specialist follow-up care rather than reducing specialist follow-up per se. In addition, given the high prevalence of current smoking in this population segment, integrating smoking cessation treatment into mental health care, rather than referral to specialist smoking cessation treatment could yield greater smoking quit success [27]. For Segment 2 patients, who had the longest elective inpatient spells and higher-than-average smoking prevalence, considering the rationing of elective surgery for procedures of limited clinical value is justifiable on prognostic grounds although a strong evidence base would be needed to conclusively establish the rationale [28].

Perhaps the most widely studied population segments are the ‘high need, high complexity’ Segments 4, 6 and 9. These Segments, which together accounted for 5% of this population and over 27% of total healthcare expenditure in the year, had the highest average number of LTCs per person. Although they are often referred to as ‘high need, high cost’ populations, their high cost consumption is probably driven by the fragmentation of care associated with the complexity of their need [29]. Consequently, a key objective for these segments should be to de-escalate need and reduce fragmentation of care by targeting integrated care management and other resources to them.

The question of how to address need and reduce use and cost of care in such high need populations merits consideration of the local determinants of need. One factor thought to drive healthcare consumption patterns of adults with multiple LTCs is the presence of functional impairment [30]. In this population however, we observed higher care consumption volumes and cost in the younger and fitter Segment 4 population compared to the older and frailer Segment 9 population. While both segments were very similar in terms of multimorbidity, their notable differences were in age (45.5 vs. 81 years), degree of functional impairment (proportion who were moderately-severely frail 28.3% vs. 66%), deprivation (88.6% vs. 78.5% in the two most deprived national quintiles) and smoking prevalence (27.2% vs. 12.8%). Despite their older age and greater functional limitation, Segment 9 patients had a lower per capita cost of care than Segment 4 patients, underlining the importance of deprivation and behavioural risk factors in driving care use (and, by extension, indicating need). This finding is consistent with those reported in other general [31] and disease-based populations [32] and implies that interventions aimed at Segment 4 patients should necessarily incorporate behaviour change support and access to broader social initiatives tackling poverty. Segment 9 on the other hand could benefit from anticipatory care planning involving both case identification and proactive intervention to reduce hospitalisation [33]. For both Segments, local health and care systems could pursue complex case management programs incorporated into or superimposed on traditional primary care systems or create specialised clinics for these Segments delivered by a multidisciplinary team offering enhanced care coordination and other support [34]. The relative merits of either approach should be explored through diverse lenses, not least of which would be capacity to engage local general practitioners and patients as well as size of potential benefit.

The approach set out in this study potentially offers a quantitative evidence base for local population health planning and delivery [35]. As segmentation processes are most useful when they iterate between quantitative and qualitative data sources [36], adding relevant qualitative social and place context to this quantitative intelligence is desirable. As a potential complement to traditional health needs assessments [37], which may lack granularity and responsiveness, this whole-population approach allows useful insight into expressed need and offers a measure of insight into non-care-seeking populations in whom unmet need may be present.

Segmenting a heterogeneous population into discrete and relatively homogenous groups with similar healthcare needs can enable the development of integrated health and care systems that are more targeted and efficient [38]. Systems that successfully achieve integration of health and care demonstrate specific attributes – chiefly (i) a focus on segments of their population with the highest need for care, and (ii) a change in core delivery processes to enable multidisciplinary teams to work around patients [39]. Segmentation and stratification of risk allows the identification of such high-risk populations and the detailed profiling of the segments based on proxies of health need potentially engages multidisciplinary teams.

Integrating health and care around segments of the population potentially tackles fragmentation of care and represents a basis for bridging the chasm in healthcare quality and outcomes often experienced by populations [4]. Achieving improved outcomes at lower cost per capita is the essence of Value-Based healthcare which depends on reliable and consistent measurement of both outcomes and cost of care in population. The potential role of population segmentation in Value-Based healthcare is evident in the fact that measurement of outcomes only works if outcomes are measured for people with similar needs.

There are potentially many other datasets which could offer greater insight into the needs of these population segments if integrated in future, for example, data from social care. Health and care policy promoting integration around patients and populations must therefore offer enabling legislative and technical environments to facilitate routine integration of datasets from diverse settings of health and care provision as well as social and demographic information.

There are potential limitations of this study worth highlighting. The creation of healthcare utilisation variables was based on an integrated primary and secondary care dataset. There are some limitations potentially associated with this approach. For example, Read codes were used to identify primary healthcare utilisation which are known to be prone to variation in their use and the overlap of different codes. In some instances, proxy measures were used where the data variables were not available. For example, GP appointment data was not available and GP practice encounters that resulted in a diagnosis Read code were taken as a proxy. Of the General Practices in the Rhondda locality, one practice did not wish to participate in the study. This practice constituted 10.3% of the GP-registered population in the locality and an element of bias may have been introduced if their practice population was significantly different to the study population. This study compared traditional segmentation with utilisation-based cluster analysis but did not look at other segmentation methodologies such as prescribed binning criteria or decision trees. There are examples in the scientific literature where these methods have achieved a greater reduction in variance than through clustering using k-means methodology [40].

Finally, it is worth placing the findings in this study in context of the degree of general deprivation in the population. Despite 60% of the population (Segments 1 and 10) using relatively little healthcare resources, the 12-month risk of emergency admission in those low-utilisation segments was 8%, a much higher rate than the 3% reported for a similar low utilisation segment in a randomly selected population in England [10]. The implications of our findings for local healthcare policy and planning may therefore differ if the population was more diverse in respect of levels of deprivation.

## Conclusion

Cluster analysis of linked primary and secondary healthcare use data for a local GP-registered population can segment the population into distinct groups with unique health and care needs. Despite some potential limitations, this approach yields valuable intelligence to inform local service planning and at the same time offers great potential for further research into its use in informing preventative, holistic health and social care.

## Data Availability

The datasets used and/or analysed during the current study are not publicly available but are available from the corresponding author on reasonable request. Access to routine administrative datasets used in the analyses was obtained from each General Practice under Data Disclosure Agreements through the NHS Wales Informatics Service.

## Abbreviations

GP: General Practice
LTC: Long Term Condition
ANOVA: Analysis of Variance
ACS: Ambulatory Care Sensitive
IHD: Ischaemic Heart Disease
COPD: Chronic Obstructive Pulmonary Disease
SD: Standard Deviation
KW: Kruskal-Wallis
ARMD: Age-Related Macular Degeneration
CHF: Congestive Heart Failure
CRF: Chronic Renal Failure
DM: Diabetes Mellitus
HT: Hypertension
ELIP: Elective In-Patient
NEIP: Non-Elective In-Patient
OPFA: Out-Patient First Attendance
OPFU: Out-Patient Follow-Up
A&E: Accident & Emergency
